# Potential of Beetroot and Blackcurrant Compounds to Improve Metabolic Syndrome Risk Factors

**DOI:** 10.3390/metabo11060338

**Published:** 2021-05-25

**Authors:** Cameron Haswell, Ajmol Ali, Rachel Page, Roger Hurst, Kay Rutherfurd-Markwick

**Affiliations:** 1 School of Health Sciences, Massey University, Auckland 0745, New Zealand; C.Haswell@massey.ac.nz; 2 School of Sport, Exercise and Nutrition, Massey University, Auckland 0745, New Zealand; A.Ali@massey.ac.nz; 3 Centre for Metabolic Health Research, Massey University, Auckland 0745, New Zealand; 4 School of Health Sciences, Massey University, Wellington 6140, New Zealand; R.A.Page@massey.ac.nz; 5 Centre for Metabolic Health Research, Massey University, Wellington 6140, New Zealand; 6 Food Innovation Portfolio, The New Zealand Institute for Plant & Food Research Ltd., Private Bag 11600, Palmerston North 4442, New Zealand; Roger.Hurst@plantandfood.co.nz

**Keywords:** anthocyanins, dietary nitrate, betalains, hyperglycemia, hypertension, dyslipidemia, diabetes, glucose control

## Abstract

Metabolic syndrome (MetS) is a group of metabolic abnormalities, which together lead to increased risk of coronary heart disease (CHD) and type 2 diabetes mellitus (T2DM), as well as reduced quality of life. Dietary nitrate, betalains and anthocyanins may improve risk factors for MetS and reduce the risk of development of CHD and T2DM. Beetroot is a rich source of dietary nitrate, and anthocyanins are present in high concentrations in blackcurrants. This narrative review considers the efficacy of beetroot and blackcurrant compounds as potential agents to improve MetS risk factors, which could lead to decreased risk of CHD and T2DM. Further research is needed to establish the mechanisms through which these outcomes may occur, and chronic supplementation studies in humans may corroborate promising findings from animal models and acute human trials.

## 1. Introduction

The term metabolic syndrome (MetS) is used to describe a group of metabolic abnormalities associated with an increased risk of coronary heart disease (CHD), cardiovascular disease (CVD), stroke, and type 2 diabetes mellitus (T2DM) [1,2]. These metabolic abnormalities include glucose intolerance, insulin resistance, central obesity, dyslipidemia and hypertension [3]. Three different definitions for MetS were in use from 2001–2009, those of the World Health Organization (WHO) [4], the Adult Treatment Panel (ATP III) [5], and the European Group for the Study of Insulin Resistance (EGIR) [6]. Each organization’s definition and measures varied slightly. Still, all agreed on the basic components of glucose intolerance, obesity, hypertension and dyslipidemia. In 2009, an international collaborative effort, including numerous health federations, developed a unified definition for MetS (Table 1) to enable and simplify data comparisons between nations and organizations [7].

As a result of the historical differences in the definitions for MetS, before 2009, it was difficult to establish and compare prevalence data, as different studies employed different measures. Despite this, in 2007, the International Diabetes Federation estimated that around one-quarter of the worldwide adult population (1.9 billion people) had MetS, and further increases were expected due to increasing obesity in developed and developing nations [8]. Furthermore, a 2007 study of MetS prevalence (using the 2001 ATP III definition) in Auckland (NZ), including 1006 Māori, 996 Pacific people, and 2020 of other ethnicities (mostly Europeans), showed that 16% of people aged 35–74 years had MetS, with higher rates in Māori (32%) and Pacific peoples (39%) [9], showing a clear difference in MetS risk between ethnic groups. Disparities between ethnicities are also shown in the US population, with non-Hispanic black women 1.2 times more likely to have MetS than non-Hispanic white women [10]. This study by Moore and colleagues [10] also showed an association between education level and MetS risk. These differences may also be explained through education and deprivation levels, with Māori and Pacific populations in NZ experiencing greater deprivation than NZ Europeans [11] and non-Hispanic black populations having a lower mean education level than non-Hispanic white populations in the US [12].

The global prevalence of T2DM rose from 108 million in 1980 to 422 million in 2014, and current projections estimate that worldwide, 591.9 million people will have T2DM by 2035 [13,14]. Early-onset of T2DM is associated with increased mortality. Data indicate that a 10 year earlier onset of T2DM results in a 1.6 times increased death rate from CVD [15]. MetS itself is associated with a higher relative risk (RR) for mortality from CVD (RR 1.74) [16]. Predictably, several studies have indicated that the presence of MetS is a significant indicator for future diabetes [2,17], with a 2013 study [18] finding a Cox proportional hazard ratio of 2.71 (95% CI 2.30–3.18). Clearly, reducing the incidence of MetS is needed to curb mortality from both T2DM and CVD.

The dietary intervention has been shown to reverse insulin resistance and T2DM [19], improve lipid profiles [20,21] and reduce cardiovascular risk [22,23]. Recent evidence suggests a role for “functional” foods and beverages containing flavonoids in reducing MetS risk [24]. Particular interest has been shown in consuming the anthocyanin subclass of flavonoids, which is associated with reduced T2DM and CVD risk [25,26,27]. Furthermore, foods high in dietary nitrate may be effective in reducing T2DM risk by improving blood glucose control [28]. In addition, dietary nitrate has been shown to lower blood pressure [29,30,31] and improve lipid profiles [32], positively impacting MetS and CVD risk factors.

While various anthocyanins are present in many common berries, blackcurrants have been shown to be a particularly good source of anthocyanins [33], a type of flavonoid responsible for the red/purple color of many berries. Anthocyanins have been shown to improve blood glucose control and other risk factors associated with MetS [34].

Common sources of dietary nitrate include leafy green vegetables and certain root vegetables. Of these, beetroot has gained renown for its unusually high nitrate content. However, it also contains high quantities of betalain pigments and other antioxidants, such as carotenoids, ascorbic acid, flavonoids, and phenolic acids [35], which may also play a role in improving risk factors for chronic disease.

Both *Beta vulgaris* (beetroot) and *Ribes nigrum* (blackcurrant) have been studied for their bioactive potential in improving postprandial blood glucose [28,36,37,38,39,40,41,42,43] and other risk factors associated with MetS [44,45,46]. This review, therefore, focuses upon and explores the current evidence for bioactive compounds in beetroot and blackcurrant and their impact on MetS risk factors. Particular focus is given to the compounds nitrate and betalains in beetroot, and anthocyanins in blackcurrant, due to their particular abundance in these foods compared to other sources, as well as the evidence for their efficacy as highlighted in the following review.

### 1.1. Bioactive Compounds in Beetroot

Beetroot contains many micronutrients in varying abundance. Beetroot contains notable quantities of thiamine (Vit. B_1_; 0.27 mg, 26% RDI), riboflavin (Vit. B_2_; 0.27 mg, 20% RDI), folate (Vit. B_9_; 80 µg, 20% RDI) and iron (0.79 mg, 9.8% RDI) per 100 g beetroot [47,48]. While these compounds are involved in many important metabolic processes, the focus of this review is on the bioactive effects of nitrates and betalains, as these compounds may have a significant effect on MetS risk factors.

#### 1.1.1. Dietary Nitrate

Beetroot stores nitrate in their roots, and along with rocket, radish and celery, are some of the foods most abundant in nitrate. The nitrate content of beetroot has been consistently recorded at over 2500 mg/kg [49,50], with values varying depending on the beetroot cultivar and growing conditions. For instance, beetroot juices prepared from 7 different cultivars showed nitrate content of between 564 ± 129 mg/L and 4626 ± 658 mg/L [50]. Furthermore, beetroot harvested in the summer contains lower nitrate concentrations than those harvested in autumn, as light causes a reduction in nitrate accumulation [51]. The nitrate content of the surrounding soil is also a key variable affecting the nitrate content of the beetroot [52], with low-soil nitrate resulting in reduced uptake into the vegetable.

Dietary nitrate may be a key bioactive within beetroot, as nitrate can be broken down into nitric oxide (NO), which plays a pivotal role in regulating vascular tone, blood pressure [30,53,54], glucose metabolism [55], and lipid peroxidation [32]. Previous studies have shown that patients with T2DM generate less NO from l-arginine compared to healthy controls [56] due to the inhibition of NO synthase by advanced glycation end products (AGEs) [57], resulting in poorer vascular tone regulation, increased blood pressure and impaired glucose metabolism.

The enterosalivary circuit acts as the pathway through which dietary nitrate is reduced into NO. Once ingested, nitrate is absorbed readily across the gut wall and transported to the blood plasma. Around 60–75% of this nitrate is then lost to excretion within 48 h of consumption [58]. Nevertheless, around 25% of nitrate in the circulation is actively concentrated by the sialin transporter to the salivary glands, where it reenters the oral cavity [59]. Here, nitrate is reduced to nitrite through nitrite-producing bacteria, such as *Staphylococcus sciuri* [60] and then swallowed before being absorbed across the gut wall to increase circulatory nitrite levels [61]. Once in circulation, nitrite is further reduced to NO by nitrite reductase [62] to function as a potent vasodilator, controlling vascular tone and blood pressure [30]. Nitric oxide also mediates glucose uptake from the intestines and skeletal muscle and could play an important role in the regulation of blood glucose levels [55].

Previous research in animal models shows that dietary nitrate supplementation increases circulating NO in animals with impaired NO production brought about by MetS [28,36]. Therefore, the high nitrate content of beetroot may offer a viable pathway to increase circulating NO in humans, particularly in groups who may have impaired NO production, such as people living with diabetes and/or MetS [57].

#### 1.1.2. Betalains

Betalains are divided into two subclasses, betacyanins, which are red pigments and betaxanthines, which are yellow pigments. Beetroot and prickly pear cacti are the only edible food products currently known to contain betalains [35]. Beetroot contains around 120 mg/100 g dry weight of betalains [63], and beetroot juice contains between 700 and 1300 mg/L of betalains depending on the cultivar and growing methods [50].

**Figure 1:**
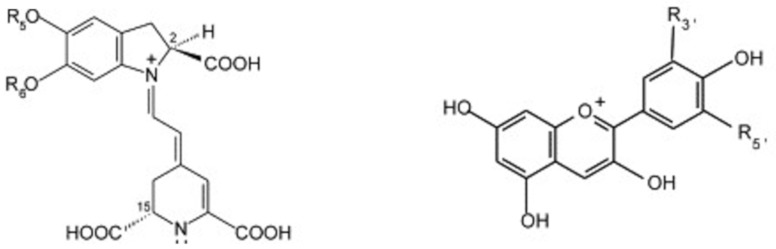
Typical structure of betalains (**left**—betacyanin) and anthocyanins (**right**—anthocyanidin).

Structurally, betalains are similar to anthocyanins (Figure 1); pigments, which give foods, such as berries their bright purple, blue and/or red colors, and within plants, betalains perform analogous functions to anthocyanins, meaning the two never naturally coexist [64]. Anthocyanins have been shown to have strong antioxidant effects, and given the similarity in structure, it seems likely that betalains will have equivalent effects. In support of this, both betacyanins and betaxanthines have been shown to have a radical-scavenging capacity three to four-fold greater than ascorbic acid, catechin and rutin [65], with other work demonstrating that the antioxidant capacity of beetroot is positively associated with betalain concentration [66].

Following beetroot juice consumption, betalain concentrations in urine are only 0.3–0.9% of that ingested [67,68], suggesting a high absorption and an alternative method of elimination, such as metabolism. Furthermore, at least 12 betalain derivatives (5 native betalains and 7 betalain metabolites) have been identified in blood plasma and urine after beetroot juice consumption [66]. Interestingly, while the betalain concentrations in blood plasma increase with beetroot consumption, after 2 weeks of chronic supplementation, the process of adaptation favoring betalain metabolism occurs, so that plasma betalain levels drop to less than 10% of that observed during the first week of supplementation [66]. This suggests that as supplementation continues, the breakdown and utilization of betalains and their metabolites increases.

### 1.2. Bioactive Compounds of Blackcurrant Juice

Blackcurrants contain many bioactive ingredients, and they are particularly potent sources of vitamin C, with just 25 g containing 100% of the Australian and New Zealand recommended daily intake (45 mg) [69]. In addition, blackcurrants contain notable quantities of potassium (322 mg; 8.5% RDI), iron (1.54 mg; 19.3% RDI), manganese (0.256 mg; 4.7% RDI) and phosphorous (59 mg; 5.9% RDI)/100 g. This review, however, focuses on the anthocyanins present in blackcurrants and their effects on markers of MetS, as these pigments are found in greater quantities in blackcurrants than most other berries and edible plants (Table 2).

While the anthocyanins listed in Table 2 are not unique to blackcurrants, they are present at higher levels than in other berries and plants, with blackcurrants containing 476 mg/100 g of anthocyanins, compared with 386 mg/100 g, 140 mg/100 g and 122 mg/100 g in blueberries, cranberries and cherries, respectively [46]. Furthermore, certain New Zealand cultivars are particularly high in anthocyanins, with the total anthocyanin content in New Zealand blackcurrant juices ranging between 346 and 850 mg/100 mL [70,71], compared to 179–310 mg/100 mL for non-New Zealand cultivars [72].

The absorption and bioavailability of anthocyanins are complex since they are highly susceptible to degradation by heat and pH variation [84]. Anthocyanins may undergo several transformations before excretion through liver microsomes and epithelial gut bacteria and via enzymatic degradation [85]. The effects of anthocyanins on health may be due to both the bioactivity of their metabolites [86] as well as their absorbed intact structures. The most abundant anthocyanins present in blackcurrants are delphinidin-3-O-rutinoside and cyanidin-3-O-rutinoside (Table 2). Röhrig et al. [87] investigated the bioavailability of these anthocyanins and their degradation products gallic acid and protocatechuic acid and found that plasma and urine concentrations peaked 2 h following ingestion, with recoveries of 0.040% and 0.048% for delphinidin-3-O-rutinoside and cyanidin-3-O-rutinoside, respectively [87]. In this study, significant quantities of bioactive degradation products were present in the plasma and urine following ingestion of blackcurrant, indicating an abundance of breakdown pathways for anthocyanins and their degradation products [87].

## 2. Effects of Dietary Nitrate and Beetroot Juice on MetS

### 2.1. Glucose Homeostasis

While investigations into the potential of beetroot juice supplementation to improve glucose control are limited (Table 3), previous studies using animal models have shown that nitrate supplementation is effective in improving blood glucose metabolism. In a placebo-controlled study, Khalifi et al. [28] divided 32 rats into 4 groups (*n* = 8): control (C), control + nitrate (CN), diabetes (D), and diabetes + nitrate (DN), and supplemented them with either sodium nitrate (CN and DN) or tap water (C and D) for 8 weeks. Before the intervention, all diabetic rats had lower serum nitrite and nitrate levels, as well as raised systolic blood pressure, compared to controls. Following 8 weeks of supplementation, in the group of diabetic rats supplemented with nitrate (DN), serum nitrite and nitrate and systolic blood pressure returned to values similar to both control groups (C and D) following 8 weeks of supplementation. Furthermore, increases in serum glucose during intravenous glucose tolerance tests for the diabetic, nitrate-supplemented rats were significantly lower than those in the diabetes control group (24.1% vs. 90.2%) [28].

Gheibi et al. [36] used a similar methodology to supplement diabetic and non-diabetic rats and demonstrated improved glucose tolerance, lipid profiles and insulin resistance during glucose tolerance tests, fasting lipids tests and insulin tolerance tests, respectively, in T2DM rats following ad libitum nitrate supplementation with sodium nitrate in drinking water. These effects were associated with decreased gluconeogenesis, inflammation and oxidative stress, and most importantly, increased expression of GLUT4 transporter proteins in insulin-sensitive tissues due to increased activation of AMP-activated protein kinase (AMPK). Before supplementation, mRNA expression and protein levels of GLUT4 were significantly lower in the soleus muscle (54% and 34%, respectively) and adipose tissue (67% and 41%, respectively) of the diabetic rats vs. controls. Supplementation increased mRNA expression and protein levels of GLUT4 translocators in the soleus (215% and 17%, respectively) and adipose tissue (344% and 22%, respectively). This is interesting, as the AMPK and GLUT4 translocation pathways are similar to those currently targeted by common antidiabetic drugs, including metformin [88].

Insulin release also causes increases in NO production, which in turn leads to the dilation of terminal arterioles to increase capillary recruitment (the number of perfused capillaries), as well as relaxation of larger blood vessels to increase peripheral blood flow [89,90]. It is thought that between 25 and 40% of glucose uptake caused by insulin release can be attributed to NO-dependent increases in blood flow to skeletal muscles [91]. As previously stated, oxidative stress from insulin resistance and glucose spikes can downregulate this response [92]. However, supplementation with dietary nitrate through beetroot juice has been shown to counteract the impairment of endothelial function typically associated with ingestion of a mixed macronutrient meal [93].

While these studies show that dietary nitrate can improve glucose metabolism in diabetic animal models, it is yet to be established if these effects can be translated to humans. Furthermore, an effective method of delivery of dietary nitrate in humans other than drinking water would be of greater significance given the lifestyle nature of MetS and T2DM. Beetroot may provide a viable and effective method of delivering dietary nitrate through lifestyle intervention.

While both beetroot juice and whole beetroot contain dietary nitrate and betalains, beetroot juice has been the preferred method of intervention in trials to date. Beetroot juice contains less dietary fiber than the whole beetroot, although dietary fiber may have beneficial effects on glucose metabolism itself [94]. However, beetroot juice does not need to be cooked, which may degrade the betalain due to heat treatment [95,96].

Five studies have investigated the effects of beetroot juice supplementation on glucose control during an acute supplementation trial [37,38,39,40,41] (Table 3). Wootton-Beard et al. [37] recruited 16 healthy volunteers to complete an oral glucose tolerance test (OGTT) with either placebo or beetroot juice (990 mg nitrate) intake in a repeated-measures design. In this study, early postprandial insulin (0–60 min) and glucose responses (0–30 min) were lowered, with a lower peak in glucose response in the beetroot supplemented (22.0 mmol/L/min) vs. the matched control (28.3 mmol/L/min). Fuchs et al. [38] recruited 16 obese, insulin-dependent patients to consume 100 mL beetroot juice (300 mg nitrate) or water but found no effect on postprandial glucose and insulin responses [38]. It is worth noting that Fuchs et al. [38] used far lower levels of nitrate supplementation (100 mL of beetroot juice containing 300 mg nitrate) than used by Wootton-Beard and colleagues [37] (225 mL beetroot juice containing 990 mg nitrate). Furthermore, Fuchs and colleagues [38] only took measures of glucose every 30 min. Hence they may not have had the sensitivity to detect differences in early phase glucose response, such as those found by Wootton-Beard et al. [37], who took measures at baseline, 5, 15, 30, 45, 60, 90, 120 and 150 min. Shepherd et al. [39] recruited 31 healthy participants to complete a 3 h OGTT with either beetroot juice (738 mg nitrate) or nitrate-depleted beetroot juice and found no differences in plasma glucose C-peptide or incretin levels. However, these measures were taken hourly, which may also have led to the same sensitivity issues as Fuchs and colleagues [38]. Furthermore, of note is that the nitrate-depleted juice acted as a control, and while depleted of the bioactive compound nitrate, this juice likely contained high betalain concentrations and therefore, both the intervention and control drinks may have had a hypoglycemic effect on glucose metabolism due to the presence of betalains.

Chang and colleagues [41] recruited 10 healthy volunteers in a crossover trial and administered either 270 mL beetroot juice (nitrate values not available) or a sugar-matched control with white bread to make 50 g of total carbohydrate. The beetroot juice intervention reduced blood glucose levels at 15, 30, 90 and 180 min compared to control, showing a reduction in the early phase and peak glucose, as well as a delay in glycemic response [41]. Furthermore, Holy and colleagues [40] administered 300 g of carbohydrate in a meal with either 250 mL of beetroot juice (nitrate not specified) or 250 mL of water and found that blood glucose levels at 2 h post-meal were significantly lowered in the test condition than in the control group.

Data from a recent longer-term supplementation study involving 30 healthy participants showed that daily consumption of a 10% beetroot juice beverage (9808 mg GAE/100 mL; volume and nitrate concentration not supplied) resulted in a 34.5% decrease in plasma glucose following 4 weeks of supplementation [97]. While these results are promising, the lack of detail in the methods for this study means that comparison is not possible, and it is difficult to draw concrete conclusions based on this research. Further research should be conducted to corroborate these findings, and interventions lasting more than 90 days would be particularly useful to determine how long-term supplementation may affect HbA1c, the gold standard measure of long-term diabetes status.

Currently, there is evidence from human trial data to suggest that beetroot juice may improve acute blood glucose responses through inhibition of salivary enzymes, delayed uptake of glucose across the intestinal wall and increased GLUT-4 translocation. While this may not lead to an improvement in incremental area under the curve during OGTT tests, there does appear to be a decreased peak in blood glucose, which may limit production of AGEs and oxidative damage associated with hyperglycemic episodes. Future studies should analyze incretins, such as gastric inhibitory polypeptide (GIP) and glucagon-like peptide-1 (GLP-1), to establish mechanisms of action or utilize interstitial glucose measurement to give a clearer picture of how these compounds are affecting glucose responses. In a chronic setting, evidence is limited to rat models [28,36] and one 6 week intervention study [97]. However, results show promise in both settings (Table 3). Chronic studies should investigate changes in HbA1C levels for examining the impact on glycemic control, and currently, dosing requirements are unclear since studies are limited and lacking detail. Dietary nitrate appears to have long-term benefits in an animal model. However, the effect of betalains is difficult to observe independently of nitrate. Studies investigating nitrate-depleted beetroot juice vs. placebo may give insight into the action of betalains on blood glucose responses and insulin sensitivity, particularly given the findings of Shepherd and colleagues [39], who found no difference between nitrate-depleted beetroot juice and normal beetroot juice on OGTT outcomes, although, as previously discussed, the sensitivity of these findings may be lower than those of Wooton-Beard and colleagues [37].

### 2.2. Hypertension

To date, several studies have investigated the effects of beetroot juice supplementation on blood pressure (Table 4), with most studies finding a significant reduction in blood pressure following both acute [31,98,99] and longer-term (1–8 weeks) supplementation [46,100,101,102,103]; however, other studies have shown no positive effects [104,105,106]. Several factors may have influenced the outcomes from these studies, including participants’ age, gender, BMI and/or beetroot dosage.

Most studies have shown that supplementation with beetroot juice has a greater effect on blood pressure in males [105,107] since premenopausal females tend to have a lower initial blood pressure than males of the same age and BMI [108]. One study showed decreased systolic blood pressure in males of 4–5 mmHg compared to 2–3 mmHg in females, 6 h after supplementation with 500 g of beetroot and apple juice (465 mg nitrate) [107]. Furthermore, a recent meta-analysis [109] showed that subjects with a BMI > 25 had greater decreases in blood pressure following beetroot juice consumption compared to subjects with a normal BMI.

The mechanisms through which beetroot supplementation may reduce blood pressure are well described, with dietary nitrate potentially increasing NO content in the vasculature through nitrate and nitrite-reducing enzyme activity. NO then stimulates cyclic 3′, 5′-guanosine monophosphate in the vascular smooth muscles, which initiates Ca^2+^ release, resulting in increased vasodilation and decreased blood pressure [52]. Interestingly, Bahadoran et al. [109], in a meta-analysis of 27 studies using nitrate-depleted beetroot juice as a control, showed that beetroot juice seems to have blood pressure-lowering effects (SBP, −2.91 mmHg: DBP −0.91 mmHg) independent of nitrate, which may suggest a role for betalains in the responses observed in previous trials.

To date, findings suggest that beetroot juice supplementation improves blood pressure regulation through increased NO content in the vasculature. Furthermore, evidence suggests there are hypotensive effects of beetroot juice independent of nitrate, meaning betalains may have an important role to play. Further investigation is needed to understand the hypotensive effect of betalains since studies to date have tended to focus on the nitrate content of their interventions and do not report betalain content. In addition, promising data from studies using anthocyanins [44,110], the structurally similar counterparts of betalains found in berries and other plants, suggests betalains may work through similar mechanisms as anthocyanins. However, these mechanisms are not presently fully understood.

### 2.3. Dyslipidemia

Different aspects of lipid profiles have been investigated following supplementation with beetroot (Table 5) in healthy individuals [40], physically fit soldiers [111] and hypercholesterolemic rats [112]. Early in vivo studies by Al-Dosari and colleagues [113] involving rats with hypercholesterolemia showed that administration of 250 mg/kg body weight of freeze-dried beetroot extract (anthocyanin data not given) significantly decreased total cholesterol and triglycerides in the intervention group vs. control. Al-Dosari and colleagues [113] also found a significant increase in high-density lipoprotein (HDL) in the intervention group vs. control.

In human trials, Holy et al. [40] showed that in healthy subjects, an acute dose of beetroot juice (250 mL, nitrate and betalains not specified) with a carbohydrate meal (300 g) lowers blood triglycerides, total cholesterol and low-density lipoprotein (LDL) [40]. Research by Singh et al. [111] involved daily supplementation of 30 soldiers with 400 mL of beetroot (nitrate and betalains not specified) juice for 15 days in a before-after study. HDL was significantly increased (1.109 ± 0.214 mmol/L to 1.298 ± 0.253 mmol/L) and LDL was significantly reduced (3.354 ± 2.128 mmol/L to 3.090 ± 2.048 mmol/L) from baseline to 15 days [111]. However, Singh et al. [111] did not include a control group, meaning conclusions may be difficult to draw as other variables, such as training status in their military rotation cycle and changes in diet due to the intervention, may have affected these results. Taken together, the results from these studies [40,111,113] show that beetroot juice may be beneficial in improving dyslipidemia both acutely and following longer-term consumption, however more robust methodology, such as randomized control trials, is needed in longer-term trials to corroborate the work of Singh and colleagues [111].

The mechanism(s) by which beetroot supplementation improves lipid profiles is not yet clear. It may be that both the increased NO production from nitrate and the potential antioxidant effects of the betalains from beetroot consumption are leading to improvements in the lipid profile. Previous work has shown that isolated betanin inhibits lipid peroxidation, leading to fewer reactive oxygen species and less oxidative damage [68]. In contrast, others [28] have shown improved lipid profiles following dietary nitrate supplementation in the absence of betalains. Combined, this may show independent effects of both betalains and nitrate in beetroot in improving lipid profiles.

It is possible that a “functional food” high in both dietary nitrate and betalains may have a marked effect on dyslipidemia in people living with MetS. Future research should focus on establishing mechanisms of action and establishing the efficacy of the intervention in a chronic trial, using a placebo-controlled crossover design. A Latin square design, including nitrate-depleted beetroot juice, may also help clarify the effects of betalains in this complex equation. Furthermore, dosing studies using different concentrations of both nitrate and betalains may aid in our understanding of the mechanisms of action through which beetroot may improve dyslipidemia.

## 3. Effects of Blackcurrant Anthocyanins on MetS

### 3.1. Glucose Homeostasis

The effects of blackcurrant anthocyanins (mostly delivered in studies as juice) on blood glucose responses have been extensively researched in recent years (Table 6), with randomized crossover and controlled trials conducted in both healthy [34,42] and overweight adults [43]. Following consumption of blackcurrant juice and blackcurrant juice fortified with crowberry powder, Törrönen et al. [114] conducted OGTT trials with 14 participants. They found that, whereas glucose and insulin responses in the early phase (0–30 min) were improved in the blackcurrant with crowberry extract group, there was no significant difference in overall AUC (area under the curve) for glucose (0–120 min). This is similar to findings in beetroot juice trials [37,41] and may indicate that carbohydrate absorption is delayed, rather than reduced, following acute supplementation.

Castro-Acosta and colleagues [34] showed that drinks containing blackcurrant extract providing 600 mg of anthocyanins effectively reduced plasma glucose responses to a high-carbohydrate meal at 0–30 min post-meal. Furthermore, plasma insulin, plasma GIP and GLP-1 were all significantly reduced following blackcurrant extract consumption compared to control. In a subsequent study [42], 25 healthy participants were supplemented with placebo, apple extract, or apple extract and blackcurrant extract in association with a starch and sucrose meal. The iAUC (incremental area under the curve; 0–30 min) improved in the apple vs. placebo trials, while apple and blackcurrant improved iAUC vs. both placebo and apple extract [42]. As apple extract has been shown to inhibit sodium-glucose transport protein-1 (SGLT-1) action [115] and proanthocyanidins in blackcurrant, inhibit alpha-amylase [116] and alpha-glucosidase [117], combining different polyphenol-rich foods may result in a synergistic effect, acting on multiple pathways, to impact on postprandial glucose responses; however further research is needed to corroborate these findings.

Watson et al. [118] took glucose measurements at 60 and 150 min following consumption of blackcurrant fruit juice (525 mg polyphenols/60 kg BW; 0.91 g carbohydrate/kg BW) following an overnight fast and observed elevated blood glucose at both time points in the intervention vs. control. While these results appear to show increased blood glucose following blackcurrant supplementation, this supports the findings of Castro-Acosta [34,42] and Törrönen [119], where the early phase blood glucose response was inhibited, with elevated glucose readings occurring at 60 and 150 min. It is possible that if glucose measurements had been taken in the early phase (0–30 min), a blunted blood glucose would have been seen at this time point. However, this can only be speculated upon. Furthermore, in this trial [118], the intervention drinks were not consumed as part of a meal but on their own, which may have affected the speed of absorption of the carbohydrates.

Several chronic supplementation studies using blackcurrant anthocyanins to investigate glucose regulation have also been conducted. Nolan et al. [43] conducted a two-experiment intervention, including an acute supplementation and a short-term (8 days) supplementation trial. They demonstrated improvements in free-living individuals’ glucose control using continuous glucose monitoring over 8 days of supplementation with a New Zealand blackcurrant extract (300 mg ingested before breakfast, 300 mg ingested before dinner; 600 mg anthocyanins per day) in overweight and obese adults [43]. Compared to placebo, insulin sensitivity was improved by 22%, and postprandial glucose responses to breakfast and lunch were reduced by 9% and 8%, respectively, in the intervention group [43].

In the acute supplementation trial, they found significant reductions in postprandial blood glucose at 45 min, 60 min and 90 min, as well as decreased AUC for glucose and insulin following acute ingestion of blackcurrant extract containing 105 mg anthocyanins with a mixed macronutrient meal [43]. These data suggest that even short-term supplementation with New Zealand blackcurrant extract can improve insulin sensitivity. This has been shown previously with blueberries [121]. However, this is the first study of its kind conducted using an anthocyanin-rich blackcurrant extract.

From the current evidence, it is clear that blackcurrant supplementation has an acute effect on blood glucose levels, delaying blood glucose responses and decreasing peak glucose during OGTTs by inhibiting carbohydrate absorption during the early phase (0–30 min). Further work needs to be undertaken to understand the appropriate doses for achieving long-term improvements in blood glucose responses, as previous work has utilized anthocyanin levels ranging from 210 mg [44] to 1200 mg [42], and have been consumed at different times of the day and at different times concerning OGTT/meal consumption.

### 3.2. Hypertension

The potential effects of blackcurrant anthocyanins on hypertension are currently not well established (Table 7). However, a recent study [44] showed that ingestion of 600 mg/day of anthocyanins from a NZ blackcurrant extract over a 7 day intervention period resulted in decreases in systolic (6 mmHg) and diastolic (6 mmHg) blood pressure in a cohort of 14 healthy older adults. Such changes are sufficient to improve health outcomes. Reducing systolic blood pressure of 6 mmHg contributes about 35–40% less stroke and 20–25% less coronary heart disease mortality [122] after just one week of supplementation.

Anthocyanins are proposed to lower blood pressure through three main mechanisms. First, anthocyanins increase endothelial-derived NO through modulation of endothelial NO synthase (eNOS) expression [123]. As previously stated, NO contributes to endothelium-dependent vasorelaxation through increased stimulation of cyclic 3′, 5′-guanosine monophosphate, in turn leading to increased blood vessel vasodilation. Second, due to their high antioxidant activity, anthocyanins can protect against reactive oxygen species, which normally interact rapidly with NO to form other radical intermediates, resulting in lower NO availability, leading to vasoconstriction and hypertension. The protective effect of anthocyanins reduces this NO conversion, thus preventing oxidative damage [124]. Third, anthocyanins are proposed to inhibit production of vasoconstriction-promoting molecules, such as angiotensin II and thromboxanes, through inhibition of the enzymes (angiotensin-converting enzyme and cyclooxygenase, respectively), which catalyze production of these vasoconstrictors [125]. Although yet to be confirmed in human trials, Park [45] also showed that blackcurrant downregulated endothelin-1 expression, another vasoconstrictor following blackcurrant supplementation in rats, and this could be another contributing mechanism to the effects observed in humans in previous trials.

While current crossover studies are limited (Table 7), the mechanisms through which blackcurrant supplementation may regulate vascular tone are well understood. More studies need to be conducted in human populations, both in an acute and chronic setting, to corroborate the findings of Cook et al. [44] and establish ideal dosage for chronic studies.

### 3.3. Dyslipidemia

As well as improving both glucose tolerance and blood pressure, consumption of blackcurrant extract also has been shown to have beneficial effects on lipid profiles in both rats [45] and human trials [119] (Table 7). Park et al. [45] induced MetS in Sprague-Dawley rats and supplemented them with placebo, 100 mg/kg/day or 300 mg/kg/day of blackcurrant extract for 4 weeks to investigate lipid profile responses. Following supplementation, rats ingesting 100 mg/kg/day and 300 mg/kg/day of blackcurrant showed significant decreases in triglyceride and LDL levels and a significant increase in HDL levels vs. control.

More recently, Nanashima et al. fed ovariectomized rats ad libitum either a regular diet or one containing 3% blackcurrant extract and showed decreased bodyweight, visceral fat weight, serum triglycerides, total cholesterol and LDL cholesterol in the extract-treated rats [126]. The findings of this study [126] show promise for the efficacy of blackcurrant anthocyanins to decrease dyslipidemia in a menopausal animal model. Postmenopausal women with low estrogen activity show an increased risk of dyslipidemia [127]. However, blackcurrant anthocyanins may attenuate this response through phytoestrogen signaling in estrogen receptors α [128] and β [129].

In human trials, Törrönen et al. [119] investigated blood glucose and free fatty acid (FFA) responses in 20 healthy females (aged 25–69 years) in a randomized, crossover, acute meal study, consuming blackcurrants or lingonberries as a puree with 35 g sucrose added or 35 g sucrose with water. Ingestion of the berry meals decreased the concentration of FFAs compared to the control meal, despite there being an overall higher carbohydrate load in the berry meal [119].

While there is evidence that blackcurrant extract supplementation can improve blood lipid profiles in an acute setting and mechanisms, including phytoestrogen signaling through receptors, chronic studies have yet to be conducted in humans, and dosages are yet to be established.

## 4. Future Perspectives

This review has focused on the efficacy of beetroot and blackcurrants to improve risk factors for MetS. While there is currently strong evidence to suggest the compounds present within these foods may have positive effects on MetS and its risk factors, some key gaps in the literature need to be addressed before it can be reliably concluded that blackcurrant and beetroot compounds improve MetS risk factors.

First, current literature investigating beetroot and MetS risk factors focuses on dietary nitrate as the active ingredient, despite evidence suggesting that betalains may have an additive or synergistic effect through other, currently unclear, pathways. It is important that future studies report both the betalain content as well as the nitrate content of interventions to ensure results can be compared between studies and to enable the sources of any observed effects to be established. Following this, studies investigating nitrate-depleted beetroot may be useful in determining the potential of betalains to affect MetS outcomes. Nitrate-depleted beetroot juice has already been suggested to improve hypertension [109]. However, further work needs to be conducted to investigate its effects on hyperglycemia and dyslipidemia.

Interestingly, research by Sawicki and colleagues [130], who supplemented volunteers with red beetroot juice for 6 weeks, found that betalain concentrations were highest in the plasma after 1 week and urine after 2 weeks. However, the lowest levels for plasma and urine were found in weeks 3 and 4, respectively. The authors suggest that this results from a process of adaptation towards betalain metabolism, as has been shown for other phytochemicals [131]. This may mean that to elicit changes in MetS risk factors, chronic supplementation with beetroot juice is more effective than acute supplementation. The shift in metabolism can only take place after 2 weeks of exposure to betalains. Trials investigating chronic supplementation should be conducted in the future, measuring plasma and urine betalain concentrations as well as MetS risk factors to establish whether this is the case.

Furthermore, in both blackcurrant and beetroot trials, there is little information regarding the appropriate doses of bioactive ingredients to elicit changes, with anthocyanin levels in blackcurrant studies ranging from 210 mg [44] to 1200 mg [42] and nitrate content administration in beetroot juice studies ranging between 68.2 mg [98] and 990 mg [37]. Future studies, particularly in areas such as hypertension, where considerable evidence currently exists, should establish appropriate minimum dosages for eliciting the improvements in MetS risk factors. This would allow more appropriate administration of any lifestyle intervention, which may increase compliance within an at-risk population.

Recent work has shown that the methods for extraction and processing of beetroot [132] and blackcurrant [133] compounds can significantly impact their betalain and anthocyanin content, respectively. Betalains [132] and anthocyanins [84] are particularly sensitive toward heat, pH, light and oxygen, leading to poor stability of these compounds during processing. Beetroot, for example, is often blanched to prolong its freshness for commercial sale, degrading the betalains [134]. This means beetroot products used in intervention trials may not be reflective of those commercially available and affordable. Recently, however, investigations into alternative processing methods have shown that high-pressure processing retains betalains and vitamin C more successfully than blanching [135]. Furthermore, studies should look to use commercially available juices and extracts when conducting trials, as these are reflective of what is applicable for the consumer. Most of the studies evaluating the effects of blackcurrant anthocyanins on biomarkers of MetS have used blackcurrants and blackcurrant extracts from New Zealand, which have reported higher anthocyanin levels [71] than non-New Zealand fruit/juice [33]. It is speculative to suggest that New Zealand blackcurrant could be more beneficial in managing MetS and its risk factors, but more research on this possibility in well-designed RCTs is required.

## 5. Conclusions

This review aimed to collect and highlight scientific evidence regarding the role of beetroot and blackcurrant consumption in the possible prevention of MetS and its associated risk factors. The current evidence demonstrates that beetroot and blackcurrant positively affects management of several MetS risk factors, including dyslipidemia, hyperglycemia and hypertension. Beetroot and blackcurrant may improve glucose uptake by activating AMPK, GLUT-4 and SGLT-1, while also inhibiting the breakdown of glucose in the intestines and saliva, leading to overall improvements in glucose control. Beetroot exhibits hypotensive effects through increasing NO availability through nitrate. However, betalains may also have a role, as has been shown for structurally similar blackcurrant anthocyanins, through modulation of endothelial NO synthase (eNOS) expression and inhibition of vasoconstriction-promoting molecule production. However, more research is required to demonstrate this conclusively. Finally, beetroot and blackcurrants may improve lipid profiles. However, the mechanisms through which this takes place are not clear, and more mechanistic as well as RCTs are needed to strengthen the currently limited evidence.

## Figures and Tables

**Table 1:** Harmonized criteria for the clinical diagnosis of metabolic syndrome (Adapted from [7]).

Criteria: three of the following five risk factors:	Diagnostic cutoff
Increased waist circumference	Country and ethnicity dependant
Raised blood pressure *	Systolic ≥ 130 and/or diastolic ≥ 85 mm Hg
Raised fasting glucose *	≥5.6mmol/L
Raised triglycerides *	≥1.7 mmol/L
Lowered HDL-C *	<1.0 mmol/L males <1.3 mmol/L females

Abbreviations: HDL-C, high-density lipoprotein. * Where drug treatment is being used to maintain healthy status, the patient is deemed to be at risk for this factor.

**Table 2:** Content of edible berries/plants for the anthocyanins delphinidin 3-O-glucoside, cyanidin 3-O-glucoside, cyanidin 3-O-rutinoside and delphinidin 3-O-rutinoside.

Anthocyanin	Plant/Fruit	Mean Content (mg/100 g FW)	Reference
Delphinidin 3-O-glucoside	Bilberry	136.0	[73]
	**Blackcurrant**	**86.68**	[74]
	Black grape	16.61	[75]
	Blueberry	15.17	[76]
	Black bean	14.50	[77]
Cyanidin 3-O-glucoside	Elderberry	794.13	[78]
	Blackberry	138.72	[75]
	**Blackcurrant**	**25.07**	[74]
	Sweet cherry	18.73	[79]
	Black olive	10.62	[80]
Cyanidin 3-O-rutinoside	**Blackcurrant**	**160.78**	[74]
	Sweet cherry	143.27	[79]
	Black olive	72.35	[80]
	Plum	33.85	[81]
	Raspberry	5.20	[79]
Delphinidin 3-O-rutinoside	**Blackcurrant**	**304.91**	[74]
	Eggplant peel	37.80	[82]
	Tamarillo	5.26	[83]

Abbreviations: FW, fresh weight. Anthocyanin contents are ordered from highest to lowest concentrations per 100 g fresh weight for each of the 4 anthocyanins. Blackcurrant values are highlighted in bold type.

**Table 3:** Details of beetroot and dietary nitrate studies investigating glucose homeostasis.

Study Author	MetS Measures	Trial Type	Subjects (*n*)	Subject Characteristics	Duration	Intervention Dosage	Placebo/Control	Outcome
Khalifi et al. 2015 [28]	Hyperglycemia, glucose tolerance	Within-groups crossover	8, 8/8	Diabetic male Wistar rats	8 weeks	100 mg/L sodium nitrate, ad libitum	Water	↓ Serum glucose ↑ Glucose tolerance
Gheibi et al. 2018 [36]	Hyperglycemia, glucose tolerance	Within-groups crossover	10, 10/10	Diabetic male Wistar rats	8 weeks	100 mg/L sodium nitrate, ad libitum	Water	↓ Serum glucose ↑ GLUT4 translocation ↑ Glucose tolerance
Wootton-Beard et al. 2014 [37]	Insulin and glucose responses	Within-groups crossover	16, 16/16	Healthy adults	Acute	225 mL BR, 990 mg nitrate	Sugar-matched placebo drink	↓ Postprandial insulin (0–60 min) ↓ Glucose response (0–30 min), ↓ glucose peak
Fuchs et al. 2016 [38]	Vascular resistance and glucose response	Within-groups crossover	16, 16/16	Obese, insulin-dependent males	Acute	100 mL BR, 300 mg nitrate	Sugar-matched placebo	↓ Postprandial vascular resistance, no effect on glucose homeostasis
Shepherd et al. 2016 [39]	Plasma glucose, C-peptide, incretins	Within-groups crossover	31, 31/31	16 Healthy young (18–35 years), 15 healthy older (50–75 years)	Acute	140 mL BR, 738 mg nitrate	Nitrate-depleted beetroot juice	No significant differences between groups
Holy et al. 2017 [40]	Blood glucose, lipid profile	Between groups, placebo-controlled	50, 30/20	Healthy adults	Acute	250 mL BR (nitrate not specified)	Water	↓ Blood glucose
Chang et al. 2018 [41]	Blood glucose	Within-groups crossover	10, 10/10	Healthy, 20–24 years	Acute	270 mL BR (nitrate not specified)	Sugar-matched placebo	↓ Blood glucose after 15, 30, 90 and 180 min
Olumese and Obah, 2016 [97]	Blood glucose	Within-groups, before–after	30	Healthy young, (aged 18–29) (18 m, 12 f)	6 weeks	10% BR (volume and nitrate not specified)	No control	↓ Plasma glucose

Abbreviations: ↓, decreased; ↑, increased; GLUT4, glucose transport protein-4; BR, Beetroot juice; m, male; f, female. Subjects are listed by total subject number then the number in each intervention group.

**Table 4:** Details of beetroot trials investigating hypertension.

Study Author	MetS Measures	Trial Type	Subjects (*n*)	Subject Characteristics	Duration	Intervention Dosage	Placebo/Control	Outcome
Coles and Clifton, 2012 [107]	Blood pressure	Within-groups crossover	30, 30/30	Healthy adults (15 m, 15 f)	Acute	500 g beetroot and apple juice (465 mg nitrate)	Apple juice concentrate	↓ SBP in males
Hobbs et al. 2013 [98]	Blood pressure, vasodilation	Within-groups crossover	24, 24/24	Healthy adults	Acute	200 g beetroot bread (68.2 mg nitrate)	200 g control white bread	↓ DBP ↑ vasodilation
Raubenheimer et al. 2017 [99]	Blood pressure	Within-groups crossover	12, 12/12	Older adults (57–71 years) (5 m, 7 f)	Acute	140 mL BR (800 mg nitrate)	Nitrate-depleted beetroot juice	↓ DBP ↓ SBP
McDonagh et al. 2018 [100]	Blood pressure	Within-groups crossover	10, 10/10	Healthy males	Acute	55 mL BR conc (357 mg nitrate)	70 mL deionized water	↓ DBP ↓ Mean arterial BP
Stanaway et al. 2019 [31]	Blood pressure	Within-groups crossover	24, 24/24	13 healthy young (18–30 years), 11 healthy older (50–70 years)	Acute	150 mL BR (651 mg nitrate)	Beetroot juice concentrate (1 mmol/150 mL nitrate)	↓ DBP in older adults, ↓ SBP in both groups
Bondonno et al. 2015 [105]	Blood pressure	Within-groups crossover	27, 27/27	Hypertensive adults (53–70 years, SBP 120–160 mmHg)	1 w	2 × 70 mL BR daily (nitrate not specified)	Nitrate-depleted beetroot juice	No significant differences between groups
Ormesher et al. 2018 [104]	Blood pressure	Between groups, placebo-controlled	41, 20/21	Hypertensive, pregnant females (SBP 130–144 mmHg and/or DBP 80–94 mmHg)	8 d	70 mL BR (400 mg nitrate)	70 mL nitrate-depleted beetroot juice	No significant differences between groups
Asgary et al. 2016 [101]	Blood Pressure, systemic inflammation	Within-groups crossover	24, 24/24	Hypertensive adults (25–68 years)	2 w	250 mL BR or 250 g cooked beetroot daily (nitrate not specified)	No control	↓ SBP ↓ DBP, ↑ FMD, ↓ Inflammatory markers
Ashor et al. 2015 [102]	Blood pressure	Between groups, placebo-controlled	21, 10/11	Overweight older adults (55–70 years, BMI 25–40 kg/m^2^)	3 w	70 mL BR conc (300–400 mg nitrate)	Blackcurrant juice (<5 mg nitrate)	No significant differences between groups
Capper et al. 2020 [46]	Blood pressure, gut microbiota	Between groups, placebo-controlled	36, 19/17	Healthy older adults	8 w	150 g whole beetroot and banana every other day (590 mg nitrate)	Banana every other day	↓ SBP

Abbreviations: m, male; f, female; ↓, decreased; ↑, increased; SBP, systolic blood pressure; DBP, diastolic blood pressure; BR, Beetroot juice; BP, blood pressure; w, weeks; FMD, flow-mediated dilation; BMI, body mass index. Subjects are listed by total subject numbers then the number in each intervention group.

**Table 5:** Details of beetroot and dietary nitrate trials investigating dyslipidemia.

Study Author	MetS Measures	Trial Type	Subjects (*n*)	Subject Characteristics	Duration	Intervention Dosage	Placebo/ Control	Outcome
Rabeh and Ibrahim, 2014 [112]	Lipid profile	Between groups, placebo-controlled	35, 7/7/7/7	Hypercholesterolemic Sprague-Dawley rats	4 w	Beetroot waste extract, 200, 400 or 600 mg/kg/day	Basal diet only	↓ TC ↓ LDL-C ↓ TG ↑ HDL-C
Khalifi et al. 2015 [28]	Hyperglycemia lipid profile	Between groups, placebo-controlled	8, 8/8	Diabetic male Wistar rats	8 w	100 mg/L sodium nitrate ad libitum	Water	↓ LDL-C ↓ TG ↑ HDL-C
Al-Dosari et al. 2011 [113]	Lipid profile, Free radical generation	Between groups, placebo-controlled	30, 6/6/6/6	Hypercholesterolemic Wistar rats	10 w	Beetroot, 250 or 500 mg/kg/day (nitrate not specified)	Basal diet only	↓ TC ↓ TG ↑ HDL-C
Holy et al. 2017 [40]	Lipid profile	Between groups, placebo-controlled	50, 30/20	Healthy adults	Acute	250 mL BR (nitrate not specified)	Water	↓TC ↓TG ↓LDL-C
Singh et al. 2015 [111]	Lipid profile	Within groups, before–after study	30, 30/0	Physically active soldiers	15 days	2 × 400 mL BR daily (nitrate not specified)	No placebo group	↓ LDL-C ↑ HDL-C

Abbreviations: ↓, decreased; ↑, increased; TC, total cholesterol; LDL-C, low-density lipoprotein cholesterol; TG, triglycerides; HDL-C, high-density lipoprotein cholesterol; BR, beetroot juice. Subjects are listed by total subject numbers then the number in each intervention group.

**Table 6:** Details of trials investigating blackcurrant juice and extracts on glucose homeostasis.

Study Author	MetS Measures	Trial Type	Subjects (*n*)	Subject Characteristics	Duration	Intervention Dosage	Placebo/Control	Outcome
Park et al. 2015 [45]	Glucose tolerance	Between groups, placebo-controlled	30, 10/10/10	Sprague-Dawley mice with metabolic syndrome	4 w	100 mg/kg/day or 300 mg/kg/day of BCE	Basal diet only	↓ iAUC glucose ↓ IRS-1 ↓ *p*-AMPK in muscle
Benn et al. 2015 [120]	Glucose tolerance	Between groups, placebo-controlled	24, 13/11	Male hypercholesterolemic rats	12 w	Equivalent 540 mg BCE in humans (actual mg unclear)	Basal diet only	↓ Plasma glucose
Törrönen et al. 2012 [114]	Blood glucose	Within-groups, crossover	14, 14/14	Healthy participants (3 m, 11 f)	Acute	300 mL BC juice and 300 mL BC juice with crowberry powder (159 and 293 mg/100 mL polyphenols)	Water	↓ Glucose response (0–30 min) ↓ Insulin response (0–30 min)
Watson et al. 2015 [118]	Blood glucose	Within-groups, crossover	36, 36/36	Healthy participants (18–34 years, BMI < 35)	Acute	Anthocyanin-enriched BCE extract or 142 mL BC juice (each containing 525 mg polyphenols)	Placebo drink (0 mg polyphenols)	↑ Blood glucose (60 and 150 min)
Castro-Acosta et al. 2016 [34]	Blood glucose insulin Incretins	Between groups, placebo-controlled	23, 23/23/23/23	Healthy participants (14 m, 9 f)	Acute	BCE, (150, 300 and 600 mg anthocyanins)	No extract	↓ Plasma glucose ↓ Plasma insulin ↓ Plasma GIP ↓ Plasma GLP-1
Castro-Acosta et al. 2017 [42]	Blood glucose-insulin incretins	Between groups, placebo-controlled	25 25/25/25	Healthy participants (20 m, 5 f)	Acute	1200 mg apple polyphenols or 600 mg apple polyphenols + 600 mg blackcurrant anthocyanins	Placebo drink (0 mg polyphenols)	↓ iAUC glucose ↓ Plasma insulin ↓ C-peptide ↓ plasma GIP
Nolan et al. 2020 [43]	Insulin sensitivity, free-living glucose, inflammation	Within-groups crossover	13 13/13	Overweight, inactive adults (BMI 28.8 ± 3.9 kg/m^−2^, <1 h structured PA/week	8 days	2 × 300 mg BC extract daily (pre-breakfast and dinner)	Placebo capsule	↑ Insulin sensitivity ↓ C-reactive protein ↓ Postprandial glucose responses

Abbreviations: w, week; BCE, blackcurrant extract; ↓, decreased; iAUC, incremental area under the curve; IRS-1, insulin receptor substrate 1; *p*-AMPK, 5′ adenosine monophosphate-activated protein kinase; m, male; f, female; BMI, body mass index; ↑, increased; GIP, glucose-dependent insulinotropic polypeptide; GLP-1, glucagon-like peptide-1; PA, physical activity. Subjects are listed by total subject numbers then the number in each intervention group.

**Table 7:** Details of trials investigating blackcurrant juice and extracts on hypertension and dyslipidemia.

Study Author	MetS Measures	Trial Type	Subjects (*n*)	Subject Characteristics	Duration	Intervention Dosage	Placebo/Control	Outcome
**Hypertension**								
Cook et al. 2020 [44]	Blood pressure	Within-groups crossover	14, 14/14	Older adults (69 ± 4 years)	1 week	2 × 300 mg BC extract daily (210 mg anthocyanins)	300 mg microcrystalline cellulose	↓ DBP↓ SBP
Okamoto et al. 2020 [110]	Arterial function, central blood pressure	Within-groups crossover	14, 14/14	Older adults (73.3 ± 1.7 years)	1 week	2 × 300 mg BC extract daily (210 mg anthocyanins)	300 mg microcrystalline cellulose	↓ Central blood pressure ↓ Carotid-femoral pulse-wave velocity
**Dyslipidemia**								
Park et al. 2015 [45]	Lipid profile	Between groups, placebo-controlled	30, 10/10/10	Sprague-Dawley mice with metabolic syndrome	8 weeks	100 mg/kg/day or 300 mg/kg/day of BC	Basal diet only	↓ TC ↓ TG ↓ LDL-C
Nanashima et al. 2020 [126]	Lipid profile	Between groups, placebo-controlled	20, 10/10	Ovariectomized female Sprague-Dawley rats	12 weeks	3% BC extract (ad libitum)	Basal diet only	↓ TC ↓ TG ↓ LDL-C
Benn et al. 2015 [120]	Lipid profile	Between groups, placebo-controlled	24, 13/11	Male hypercholesterolemic rats	12 weeks	Equivalent 540 mg BC extract in humans (Actual mg unclear)	Basal diet only	↓ TC
Törrönen et al. 2012 [119]	Glucose, insulin, free fatty acids	Within-groups crossover	20, 20/20	Healthy females	Acute	150 g BC puree with 35 g sucrose (anthocyanins not specified)	Water	↓ FFA rebound following a meal

Abbreviations: w, week; BC, blackcurrant; ↓, decreased; ↑, increased; DBP, diastolic blood pressure; SBP, systolic blood pressure; TC, total cholesterol; TG, triglycerides; LDL-C, low-density lipoprotein cholesterol; FFA, free fatty acids. Subjects are listed by total subject numbers then the number in each intervention group.
